# The Application of Auxin-like Compounds Promotes Cold Acclimation in the Oilseed Rape Plant

**DOI:** 10.3390/life12081283

**Published:** 2022-08-22

**Authors:** Jurga Jankauskienė, Rima Mockevičiūtė, Virgilija Gavelienė, Sigita Jurkonienė, Nijolė Anisimovienė

**Affiliations:** Laboratory of Plant Physiology, Nature Research Centre, Akademijos Str. 2, 08412 Vilnius, Lithuania

**Keywords:** auxin-like compounds, cold acclimation, dehydrins, IAA metabolism, oilseed rape

## Abstract

Cold is a major environmental key factor influencing plant growth, development, and productivity. Responses and adaption processes depend on plant physiological and biochemical modifications, first of all via the hormonal system. Indole-3-acetic acid (IAA) plays a critical role in the processes of plant functioning. To assess the influence of the auxin-like compounds 1-[2-chloroethoxycarbonylmethyl]-4-naphthalenesulfonic acid calcium salt (TA-12) and 1-[2-dimethylaminoethoxycarbonylmethyl]naphthalene chloromethylate (TA-14) in the process of cold acclimation, long-term field trials over four years were performed with two rapeseed (*Brassica napus* L.) plant cultivars with different wintering resistance in temperate-zone countries. In these two rapeseed cultivars, namely ‘Casino’ (less resistant) and ‘Valesca’ (more resistant), investigations were conducted in the terminal buds and root collars. The application of auxin-like compounds revealed a close interlinkage between the composition of dehydrins and the participation of the phytohormone IAA in the adaptation processes. By applying TA-12 and TA-14, the importance of the proteins, especially the composition of the dehydrins, the IAA amount, and the status of the oilseed rape cultivars at the end of the cold acclimation period were confirmed. Following on from this, when introducing oilseed rape cultivars from foreign countries, it may also be of value to assess their suitability for cultivation in temperate-zone countries.

## 1. Introduction

In preparation for wintering, cold acclimation by plants is a multiple biochemical-physiological process that is typical for biannual and perennial herbaceous temperate climate plants [1,2,3]. The genetic determination of how that process is anticipated by genus, species, or even cultivars is under investigation [3]. It was found that during acclimation to low temperatures, triggered by the influence of short days, plants acquire resistance to unfavorable temperatures (cold) and temperature fluctuations in winter [4,5,6,7,8]. The main environmental factors that induce the intracellular mechanisms of plant acclimation in herbaceous plants are a regime of low positive temperature (from +10 °C to +2 °C) and changes in photoperiodicity, i.e., the shortening of the day [6,9,10,11]. The modification of biochemical-physiological processes in plant tissues and changes of physical-chemical properties in cell membranes are observed during the plant cold acclimation period [11,12,13,14].

Although the role of the hormonal regulatory system in these processes is indisputable, the main focus for research so far has been on the inhibitory phytohormone ABA [5,15,16,17,18,19]. Despite the fact that IAA is considered the main phytohormone participating and controlling most of the physiological processes [20], with it being the trigger of morphological reorganization at the cell and the whole plant level [21], there are few publications on the role of the fund transformation of this phytohormone in plant autumn growth nor of its relationship to cold acclimation development [22,23]. Furthermore, usually experiments are carried out in model systems for research into cold acclimation mechanisms, i.e., simulating the temperature mode and a light period that is typical for the cold acclimation period. It has been shown that such experiments cannot be directly compared with the cold acclimation process that is taking place in plants that are growing under natural conditions in the wild [24,25].

The most basic indicators for the preparation of the cold acclimation period are considered to be changes in the genetic or protein composition [23,26]. According to the opinions of researchers who analyzed plant biochemical and physiological changes during the cold acclimation period under natural conditions, the only good cold acclimation of biennial or perennial plants that acquire resistance to adverse environmental factors were those that were able to maintain the metabolic processes that is typical for the dormancy period [4,17,27]. The protein composition and the possible value of the condition of phytohormones and their physiological activity for the property of plant cold acclimation was highlighted in these experiments [28,29]. To summarize, natural habitat plants acclimate completely, while crops, especially those originating from warmer climatic zones, separate species, or even their individual cultivars, do not often successfully overwinter [29,30]. There is no straight answer to what determines the plant property for preparing properly for cold acclimation and its resistance to adverse environmental factors [30,31].

The synthetic auxin-like compounds 1-[2-chloroethoxycarbonyl-methyl]-4-naphthalenesulfonic acid calcium salt (TA-12) and 1-[2-dimethylaminoethoxicarbonylmethyl]naphthalene chlormethylate (TA-14) are similar in structure to naphthyl acetic acid. An analysis of the structure and activity of these auxin-like compounds has shown that the activity of the compounds TA-12 and TA-14 is determined by the two methylene groups in their molecules, as well as a sulfo group that is attached to the naphthalene ring (TA-12) and a methyl group (CH_3_) that is attached to N^+^ (TA-14). Our previous study showed the effect of auxin-like compounds on the accumulation of proline and saccharides in the root collar and terminal bud in oilseed rape and, furthermore, that these compounds influence the acclimation of plants to the cold, overwintering, and productivity formation [32]. However, from a theoretical point of view, these results also raised a question regarding which metabolic processes may also be integrated into the metabolism of the cell systems during the cold acclimation period, successfully overwintering, and maintaining dormancy in plants. In the recent study, it was shown that plant adaptability and cold acclimation is one of the most important physiological, biochemical, and biotechnological problems for plants. Moreover, the detection of metabolic processes that are responsible for the mechanisms of resistance/adaptability in plants remains important, along with experiments on their wintering improvement with biotechnology tools [30,33,34,35,36,37,38]. Physiological-biochemical peculiarities of plant cold acclimation-preparation for wintering under natural conditions, including protein composition and especially changes in the amount and the status of the hormonal-regulatory systems of the phytohormone IAA under exogenous auxin-like compounds, have not been investigated or disclosed to date.

Oilseed rape is the most popular crop in Europe and it is used in many industry areas, including food. European Union countries are the most important producers of the oilseeds [39]. Climate change, mainly due to temperature fluctuations, is projected to significantly limit oilseed production growth [40,41].

The main tasks of our study were to investigate the protein composition, the amount of phytohormone IAA and the characteristics of its changes in state in organs that were significant for the wintering (terminal bud and root collar) of two oilseed rape (*Brassica napus* L.) cultivars with different wintering resistances in natural field conditions during the cold acclimation and preparation for wintering period. By applying auxin-like compounds, peculiarities of adaption to cold processes were also investigated.

## 2. Materials and Methods

### 2.1. Object of Research

Organs that were important for wintering—the terminal bud and root collar—of two different oilseed rape cultivars (*Brassica napus* L. ssp. *napus*), i.e., ‘Casino’ (less resistant to wintering) and ‘Valesca’ (more resistant to wintering) were selected as the test objects for the investigation of protein composition, the amount of phytohormone indole-3-acetic acid (IAA), and the changes of state during the plant preparation for the wintering (dormancy) period.

### 2.2. Field Experiments

Oilseed rapes were grown in small fields of one square meter plots in three replications at the Field Experimental Station (54°68′ N, 25°26′ E) of the Institute of Botany, the Nature Research Centre (Lithuania). The main agrochemical parameters of the arable soil layer were pH 7.0–7.3, P_2_O_5_ 248–250 mg/ha, and K_2_O 214.0–214.6 mg/kg. Following the common cultivation practices for sowing and fertilization, the test plants were sown in the third week of August [42]. The row spacing of oilseed rapes was 12.5 cm; the winter oil seeds were placed at 2–3 cm depth. The density of rape seeds in autumn was 80–100 plants/m^2^. For the evaluation of the biometric characteristics, protein composition, IAA amount, and its fund composition during the cold acclimation period, plants were taken for investigations during the autumn growth at the BBCH-14 phenological growth stage and then periodically over the whole cold acclimation period: specifically during the early cold acclimation period (mid-October, average daily temperature +10 °C); during the late cold acclimation period (end of October, average daily temperature +5 °C); and at the end of the cold acclimation period (first week of November, average daily temperature 0 °C and below).

The auxin-like compounds 1-[2-chloroethoxycarbonylmethyl]-4-naphthalenesulfonic acid calcium salt TA-12 and 1-[2-dimethylaminoethoxycarbonylmethyl]naphthalene chloromethylate (TA-14) [43] were used for the research into the changes in the amounts of endogenous phytohormone IAA and its conditions in order to investigate the peculiarities of adaptation to cold acclimation processes and the relationship of protein composition with wintering at the end of the cold acclimation process. The test plants were sprayed with solutions of aqueous auxin-like compounds TA-12 (2 mM) and TA-14 (4 mM) at the BBCH-13 phenological growth stage. Plants that were affected by these compounds were investigated along with control plants assessing the IAA amount, status, and protein composition at the end of cold acclimation period.

### 2.3. Extraction of Easily Soluble Protein (ESP) and Heavily Soluble Protein (HSP) Fractions and Thermostable Proteins

Fractions of ESP (buffer-soluble, mainly cytosolic and apoplast proteins) and HSP (proteins from cell membrane components) were extracted according to the methodology that was used in the laboratory, customized for easily (extractable using buffer) and hardly (extractable only with detergent additive) soluble fractions and suitable for electrophoretic analysis of cell proteins from the vegetative organs of oilseed rapes test cultivars in the terminal bud and root collar [44,45].

A total of 100 mM Tris-HCl buffer (pH 8.3) was used for the extraction of the ESP and HSP fractions: 1 mM EDTA, 1 mM PMSF, and 1 mM DTT for the ESP fraction with stabilizing components, 100 mM Tris-HCl buffer (pH 8.3) with 1% non-ionic detergent Triton X-100 for the HSP fraction. The protein fractions were extracted at +4 °C temperature with a ratio of fresh material to buffer of 1:3. For the extraction of each fraction, 3–4 consequent extractions until isolation of one or another protein fractions were performed. The protein fractions were separated by centrifugation (10,000× *g* 10 min) (cooling centrifuge PMW-35, Warsaw, Poland). After the full three stages of ESP extractions from the experimental material, the next stage was extraction of the HSP fraction. The extracted solvents from the three stages of extraction from the same protein fractions were combined. Precipitation with organic salts (ammonium sulfate up to 85% saturation) along with gel filtration multistage chromatography on Sephadex G-25 (Merck KGaA, Darmstadt, Germany) was used for the cleaning of the ESP preparations. XAD-7 was used for the removal of surplus detergent Triton X-100 from the HSP fraction. Preparations of the ESP and HSP factions were used for assessing the protein composition during the subsequent stages of the investigation.

Thermostable proteins were extracted from the preparations of the ESP and HSP fractions by heating them at +70 °C or 100 °C temperature [45]. Later, a fraction of the thermostable proteins was extracted from the supernatant, chilled at +4 °C temperature for 30 min, and separated by centrifugation (30,000× *g* 60 min) (VAC-601, Leipzig, Germany).

The protein amount in the identified fractions and all the subsequent stages of the investigation was evaluated using the Bradford method at a wavelength 595 nm [46] and measured by a spectrophotometer SPECORD 210 Plus (Analytik Jena GmbH, Jena, Germany). Bovine serum albumin (monomer, 66 kDa) was used as the standard.

### 2.4. Analysis and Identification of Protein Preparations Using Electrophoresis Methods

The protein composition was tested using polyacrylamide gel electrophoresis methods—native PAGE and SDS-PAGE [47]. The total ESP and HSP fractions and the composition of thermostable protein were analyzed using PAGE and polypeptides—SDS-PAGE.

The concentration of the starting gel was 4%, the separating gel was 10%. The protein amount that was placed in one track was 50 µg. The distribution of the proteins using both PAGE and SDS-PAGE was carried out using ProteanII xi Cell (Bio-Rad, Hercules, CA, USA) and Multigel-Long (Biometra, Göttingen, Germany) apparatus, 10 mA for the starting gel, and 25 mA for the separating gel. Gels were visualized using Coomassie brilliant blue R250.

Molecular masses were estimated according to the localization in electrophoresis gels using standards: for non-denaturing electrophoresis, we used a Molecular Weight Marker Kit, 14,000–500,000 range (Sigma, JAV, St. Louis, MA, USA); for denaturing electrophoresis, we used Precision plus Protein Standards All Blue, 10,000–250,000 range (Bio-Rad, JAV).

### 2.5. Identification of Dehydrins Using Immunoblotting Method

The transportation of polypeptides from gels onto nitrocellulose membranes (diameter of pores 0.45 µm, Bio-Rad, Hercules, CA, USA) was performed using semi-dry blotting (Biometra, Göttingen, Germany), 1 mA cm^−2^ for 1.5 h. Transfer buffer: 48 mM Tris-HCl, pH 9.2; 39 mM glycine, 20% methanol, 0.0375% SDS, pH 9.6 [47].

The membrane was blocked by a buffer for 1 h (10 mM Tris, 500 mM NaCl, pH 7.4) with an additive of non-fat dry milk powder. The buffer for washing TTBS: 20 mM TBS, with 0.05% of Tween-20. The membrane with the antibody buffer (using TTBS, 1.5% non-fat milk dry powder, anti-dehydrins antibodies (1:1000)) [48] was incubated overnight. After the washing procedure with TTBS for 10 min, the membranes were incubated with the secondary antibodies (1:3000) for 2 h (GAR-AP, Bio-Rad, USA). After the last wash using TTBS and TBS (each for 10 min), the membranes were highlighted with a color reagent (AP Conjugate Substrate, Bio-Rad, USA) with a buffer. Colorimetric evaluation was performed using a calibrated densitometer (Molecular Imager GS-800TM USB Calibrated Densitometer, Bio-Rad, USA).

### 2.6. Extraction of Free IAA and IAA Conjugates and Other Metabolites

Commonly used methods, experimental techniques, and procedures were used for the extraction of indole compounds from the fresh material and their characterization [49]. Indole compounds from the terminal buds and root collars were extracted using 80% cold methanol with antioxidant BHT (10 mg L^−1^), in the dark at +4 °C for 14 h. The ratio of extrahent and material was 1:10. The methanolic extract of the plant material was separated by filtration through a nitrocellulose membrane, the pore size was 0.2 µm (Whatman, Maidstone, UK); phenolic compounds were removed with PVPP. The extract was concentrated using a vacuum evaporator (IKA RV 10 Basic, Staufen, Germany) into the aqueous residue or until dry, depending on the experimental goals.

Standard fractionation procedures under different pH and solubility in different organic solvents were applied for the analysis of indole compounds: extracted indoles soluble in ether, at pH 3.0; in butanol, at pH 2.5; in ethylacetate, at pH < 3.0 fractions [50]. The alkaline hydrolysis method was applied for the elucidation of IAA from the IAA conjugates complexes: IAA release from the IAA-ester complexes—1 N NaOH, at +30 °C for 30 min and IAA-amide complexes—7 N NaOH, at +100 °C for 1 h [49]. From the complexes that were released, IAA was extracted using ether.

### 2.7. Characterization and Identification of IAA and Its Conjugates

Preliminary identification of indole compounds was performed using the TLC method. The prepared preparations were applied on Alugram SIL G/UV254 plates (Macherey-Nagel, Düren, Germany). There were two different chromatography systems that were used (acidic—n-butanol: glacial acetic acid: water (4:1:4) and alkaline—isopropanol: ammonia: water (8:1:1)). The fluorescence of the indole compound spots was determined at a wavelength λ254 nm and the localization place (Rf) was calculated according to the ratio of the solvent’s finish and the spots’ center lengths. Color reactions were carried out with reagents Salkowski and Erlich [51]. The absorption spectrums of IAA and its metabolites were determined by the spectrophotometer (SPECORD 210 Plus, Germany), with the measuring absorption in the range of λmin200–λmax320 nm [49]. The indicators of endogenous indole compounds were compared with standard indicators of synthetic indole compounds. The following standards were used for the research: IAA, IAA-amide—IAA-Val and IAA-Ala, IAA-Asp, IAA-Glu, IAA-Leu, IAA-Phe; ester—IOxGlc; 4-Cl-IAA, and catabolites—5OHIAR, oxIAR (Olchemim, Olomouc, Czech Republic).

The amounts of indole compounds were calculated using a calibration curve, which was prepared based on the known concentrations of the IAA standards.

The final identification of IAA, its conjugates, and other metabolites was carried out using the HPLC method [52]. A high-pressure liquid chromatograph Shimadzu CTO-20A/20AC (Kyoto, Japan) with reversed-phase column C18 (15 cm × 4 cm) was used for this purpose. The diameter of the sorbent particles was 3 μm. The methanol gradient elution was from 5% to 99% using eluent system methanol: water: acetic acid. The eluent flow rate was 0.6 mL/min^−1^, the volume of material that was injected was 20 μL. UV detection was carried out at the wave length of 280 nm. IAA and its conjugates were identified by the retention time at the column (min) in comparison to the standards of their absorption spectra.

### 2.8. Statistical Analysis

The experimental data were analyzed using STATISTICA for Windows version 11. The results of the biochemical and biometrical parameters represent data that are expressed as the means of determinations that were made in triplicates and tested by ANOVA, followed by comparisons of means by the Duncan test *p* < 0.05. Arithmetic means ± standard deviations (SD) were calculated from four biological experiments that were carried out under natural conditions.

## 3. Results

### 3.1. Changes in the Number of Proteins during the Autumn Growth Period and the Various Stages of Cold Acclimation

**Figure 1:**
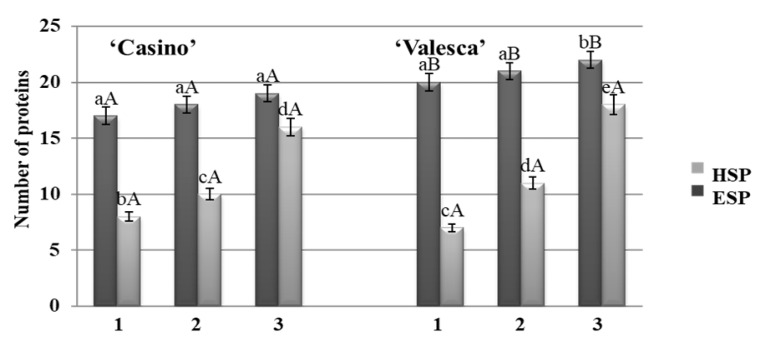
Number of ESP and HSP proteins in the terminal buds of the oilseed rape cultivars ‘Casino’ and ‘Valesca’. 1—autumn growth period; 2—early cold acclimation period; 3—end of the cold acclimation period. There were four biological experiments with three replicates in each. Within each cultivar, different lowercase letters indicate significant differences in the mean number of proteins in the same fraction (either HSP or ESP) among the three different growth and cold acclimation periods. Different uppercase letters indicate significant differences in the mean number of proteins in the same fraction between the two rapeseed cultivars. Bars are the means ± SD with different letters and are significantly different at *p* < 0.05 according to the Duncan test.

During the analysis, no differences were observed in the number of proteins that were detected by native PAGE in the terminal buds of either oilseed rape cultivars during the autumn growth periods (Figure 1(1)). However, differences with respect to the cultivars, organs, and fractions of proteins were revealed during the process of cold acclimation period. A comparative analysis of protein composition and their changes in both oilseed cultivars in their early cold acclimation period revealed a general trend of increasing numbers of proteins (Figure 1(2)).

**Figure 2:**
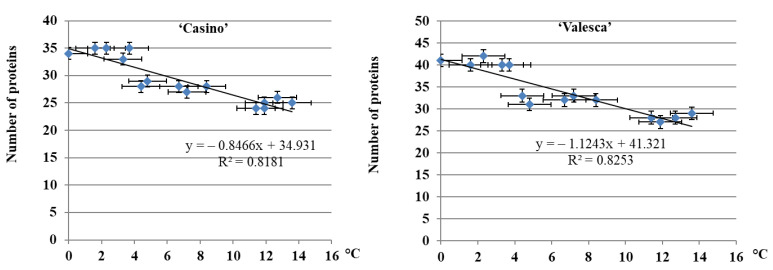
Dependence of the number of proteins in the terminal buds of the oilseed rape cultivars ‘Casino’ and ‘Valesca’ on the average daily temperature. There were four biological experiments with three replicates in each. All spots indicate the means ± SD.

The reliability of the results was confirmed by correlation-regression data analysis. After having calculated the dependence of the number of proteins that were detected in the terminal bud of both plant cultivars through the duration of cold acclimation, it was found that the number of proteins increased statistically reliably in both fractions, while the average daily ambient temperature decreased. This correlation was confirmed by the determination coefficient (R^2^), R^2^ = 0.81 for ‘Casino‘ plants and R^2^ = 0.82 for ‘Valesca’ plants (Figure 2).

**Figure 3:**
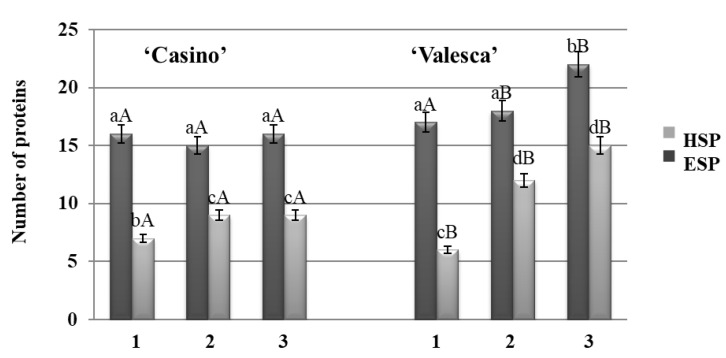
The number of ESP and HSP proteins in the root collars of the oilseed rape cultivars ‘Casino’ and ‘Valesca’. 1—autumn growth period; 2—early cold acclimation period; 3—end of the cold acclimation period. Four biological experiments with three replicates in each. Within each cultivar, different lowercase letters indicate significant differences in the mean number of proteins in the same fraction (either HSP or ESP) among the three different growth and cold acclimation periods. Different uppercase letters indicate significant differences in the mean number of proteins in the same fraction between the two rapeseed cultivars. Bars are the means ± SD with different letters and are significantly different at *p* < 0.05 according to the Duncan test.

No significant differences were found in the root collars of the two protein fractions in either cultivar during the autumn growth. The total number of proteins that was detected was the same—23 (Figure 3). No deviation in the number of proteins in the root collar of the cv. ‘Casino’ was detected, whereas the composition of proteins that were detected in the cv. ‘Valesca’ changed greatly and intensively: even to the extent that six (6) proteins were newly formed in the HSP fraction (Figure 3).

Significant changes in the composition of the proteins in the root collar were observed in the more resistant to wintering cv. ‘Valesca’ by the end of the cold acclimation period, with the increased number of newly formed proteins being up to seven (7), while only one (1) was formed in the ESP fraction of cv. ‘Casino’ (Figure 3). No significant changes in the composition of the HSP fraction proteins in the root collar were observed in the less resistant to wintering cv. ‘Casino’ during the same period.

**Figure 4:**
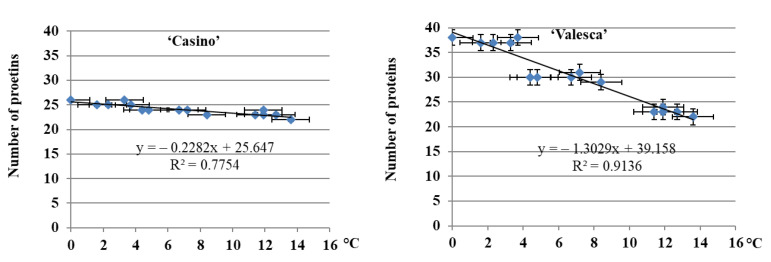
Dependence of the number of proteins in the root collars of the oilseed rape cultivars ‘Casino’ and ‘Valesca’ on the average daily temperature. There were four biological experiments with three replicates in each. All spots indicate the means ± SD.

The reliability of the results was confirmed by the correlation-regression data analysis (Figure 4).

After having calculated the dependence of the number of proteins that were detected in the root collars of both plant cultivars upon the duration of cold acclimation, it was found that the number of proteins increased statistically reliably in both fractions, while the average daily ambient temperature decreased. This correlation was confirmed by the determination coefficient (R^2^): R^2^ = 0.77 for cv. ‘Casino’ plants, R^2^ = 0.91 for ‘Valesca’ plants (Figure 4). However, unlike cv. ‘Valesca’, no linear correlation of both numbers of the protein fractions in the root collars of cv. ‘Casino’ depending on the ambient average daily temperature was detected (Figure 4). The results showed that the transformation of proteins in the root collars of cv. ‘Valesca’ was more intensive than compared to cv. ‘Casino’.

### 3.2. Characteristics of the Differing Protein Metabolic Processes in the Oilseed Rape Cultivars According to Their Resistance to Wintering at the End of Cold Acclimation Period

Significant changes in the protein number, this being an increase in their total accumulation during the whole cold acclimation period, is relevant for the more resistant to wintering cv. ‘Valesca’ in comparison to the less resistant cv. ‘Casino’ (Table 1). Especially evident was the increase in the number of newly formed proteins in the root collar of cv. ‘Valesca’.

At the end of the cold acclimation period, 25.80% of the proteins that were characteristic of the autumn growth period had disappeared from the terminal bud of the oilseed rape cv. ‘Valesca’ This is a lower level of disappearance than in the cv. ‘Casino’ where the comparable figure was 31.15%. The number of proteins that remained during the entire cold acclimation period in the apical bud of cv. ‘Valesca’ was 36.95% of the total cell protein, which was significantly higher than that of the cv. ‘Casino’. In addition, the newly formed protein at the end of cold acclimation period, not typical for the autumn growth period, in the root collar of the cv. ‘Casino’ was 32.25%, but in cv. ‘Valesca’ this was 38.12%. For the oilseed rape root collar of the cv. ‘Valesca’, 45.03% of the proteins were the same throughout the cold acclimation period. The number of proteins remaining during the entire cold acclimation period in the root collar of cv. ‘Valesca’ was 45.03% of the total cell protein, in cv. ‘Casino’ was lower at 42.50% (Table 1).

In terms of protein metabolism during the cold acclimation period, the more resistant oilseed rape cv. ‘Valesca’, which retains a number of proteins that are typical for the autumn growth period, was considered more stable; and more new proteins were formed, especially in the root collar. The total number of proteins is higher during the whole cold acclimation period and at the end of it having transformed into the dormancy period.

### 3.3. Peculiarities of the Changes in Proteins Composition of the Two Oilseed Rape Cultivars That Differ According to Their Resistance to Wintering

**Figure 5:**
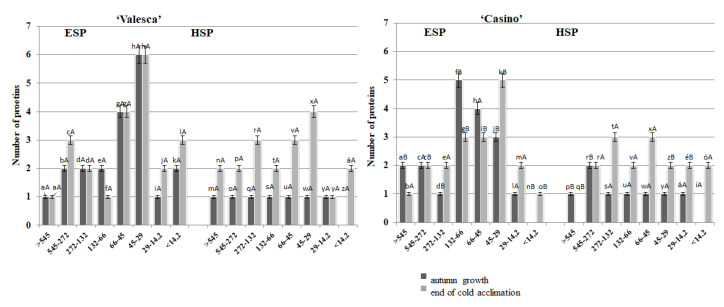
Composition of the ESP and HSP fractions in the terminal buds of the oilseed rape cultivars ‘Valesca’ and ‘Casino’ during the autumn growth and at the end of the cold acclimation periods. There were four biological experiments with three replicates in each. Within each cultivar, different lowercase letters indicate significant differences in the mean number of proteins at each segment in the same fraction (either HSP or ESP) in the growth and cold acclimation periods. Different uppercase letters indicate significant differences in the mean number of proteins in each segment in the same fraction between the two rapeseed cultivars. Bars are the means ± SD with different letters and are significantly different at *p* < 0.05 according to the Duncan test.

**Figure 6:**
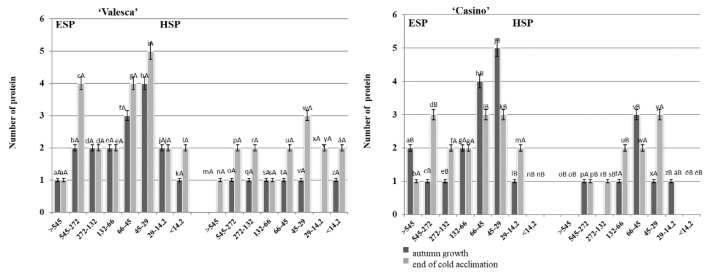
Composition of ESP and HSP fractions in the root collars of the oilseed rape cultivars ‘Valesca’ and ‘Casino’ during the autumn growth and at the end of the cold acclimation periods. There were four biological experiments with three replicates in each. Within each cultivar, different lowercase letters indicate significant differences in the mean number of proteins in each segment in the same fraction (either HSP or ESP) in the growth and cold acclimation periods. Different uppercase letters indicate significant differences in the mean number of proteins in each segment in the same fraction between the two rapeseed cultivars. Bars are the means ± SD with different letters and are significantly different at *p* < 0.05 according to the Duncan test.

Native PAGE data revealed that the largest numbers of ESP proteins in the two organs, from 14.2 kDa to 66 kDa, in both oilseed rape cultivars during the autumn growth period were accumulated at the end of the cold acclimation and during the transition to the dormancy period. A significant increase in the number of the medium and low molecular weight proteins was observed during the cold acclimation period (Figure 5 and Figure 6). This was particularly evident for the increased number of proteins from 29 kDa and the lower molecular weight proteins in the ESP fractions of both oilseed rape cultivars.

An increase in the number of proteins was observed in the HSP fraction of the terminal buds in both oilseed rape cultivars. No proteins were found in the HSP fraction of the terminal bud in the zone of macromolecular proteins in the cv. ‘Casino’ at the end of the cold acclimation and the transition to the dormancy period (Figure 5), whereas two proteins were found in the same zone in the cv. ‘Valesca’. The most significant increase in three proteins that were not typical for autumn growth that were detected in this zone was observed in the range of 45 to 29 kDa, whereas only one protein was found in the cv. ‘Casino’. An increase in two proteins in both cultivars was observed in the range of 14.2 kDa and lower molecular weight.

The same increasing trend in the number of proteins was detected in the root collar during the cold acclimation as was in the terminal bud, i.e., the largest changes occurred not only at the medium and lower protein zones but were accumulated exactly in these zones at the end of the cold acclimation period (Figure 6).

### 3.4. Number of Polypeptides in the Oilseed Rape Cultivars with Different Resistance to Wintering during the Cold Acclimation Period

An analysis of the changes in the number of polypeptides using SDS-PAGE, different from native PAGE, when proteins retain their native structure and functions, was carried out during the second level of the proteins study in an attempt to highlight the specificity of changes in their composition during the cold acclimation period in the organs of the oilseed rapes—i.e., the terminal buds and root collars.

The number of polypeptides in the ESP fraction of the cv. ‘Valesca’ terminal bud increased twice during the acclimation period. At the same time, the number of polypeptides in the cv. ‘Casino’ increased less, specifically 1.65-fold (Table 2). The number of ESP fraction polypeptides increased 1.5-fold in the root collar of both oilseed rape cultivars at the end of the cold acclimation period.

According to the research results, twice fewer polypeptides in the HSP fraction of the terminal bud and root collar of the less wintering-resistant cv. ‘Casino’ were detected compared to the more wintering-resistant oilseed rape cv. ‘Valesca’, i.e., 51.31% and 62.46%, respectively.

A much higher number of individual polypeptides was detected in cv. ‘Valesca’ comparing to the cv. ‘Casino’ at the end of the cold acclimation period. Furthermore, polypeptides that were smaller than 25 kDa were detected only in the cv. ‘Valesca’ cv., whereas none were detected in the ‘Casino’ cv. during the entire investigation period.

### 3.5. Characterization of Thermostable Proteins Found in the Oilseed Rape Cultivars Different for Their Resistance to Wintering during the Cold Acclimation and Preparation to Wintering Period

The comparison of the electropherograms of the thermostable protein ESP and HSP fractions that were extracted from the terminal buds and root collars of the oilseed rape cultivars ‘Casino’ and ‘Valesca’ revealed that the protein composition of the ESP and HSP fractions also caused changes in the composition of the thermostable proteins in the organs of both of the investigated winter oilseed rape cultivars during the cold acclimation and preparation for the wintering period. The longer duration of the cold acclimation period caused an increase in the number of thermostable proteins in the investigated organs of both studied cultivars (Table 3).

When considering the percentage of thermostable proteins in the total number of proteins, more intensive changes were observed in the ESP fractions of both organs. During the autumn growth period, the number of thermostable proteins in the ESP fractions in the root collar of cv. ‘Valesca’, and especially in the terminal bud, were significantly (statistically reliable) higher than in those of the cv. ‘Casino’ (Table 3). At the end of the cold acclimation period, the percentage of thermostable proteins in the ESP fraction increased in both organs in both cultivars that were studied. Significantly higher numbers were detected both in the terminal bud and in the root collar in the more resistant to wintering ‘Valesca’ than in the less resistant to wintering cv. ‘Casino’ (Table 3). No statistically significant differences according to the number of proteins were detected in the HSP fractions of either oilseed rape cultivars (Table 2).

### 3.6. Identification of the Specific Thermostable Proteins-Dehydrins by the Western Blotting Method

**Figure 7:**
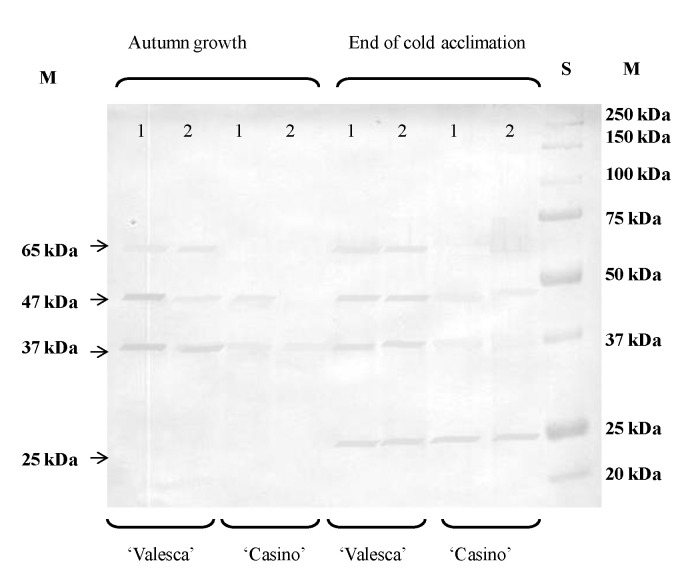
Composition of dehydrins in the terminal buds (1) and root collars (2) of oilseed rape cultivars ‘Casino’ and ‘Valesca’ during the autumn growth and at the end of the cold acclimation period.

There were three dehydrins, specific thermostable proteins that determine plant resistance to cold, that were identified during the autumn growth period and four dehydrins at the end of the cold acclimation. There were 37, 47, and 65 kDa dehydrins that were identified in the terminal bud and root collar of the cv. ‘Valesca’ during the autumn growth period (Figure 7(1)). However, the 65 kDa dehydrin was not identified in either of the investigated organs in the cv. ‘Casino’ and 47 kDa was only in the root collar (Figure 7(2)). The newly formed 25 kDa dehydrin, not typical for the autumn growth period, was identified in both organs of the investigated oilseed rape cultivars at the end of the cold acclimation period. The 37 kDa dehydrin was significantly accumulated in the root collar of cv. ‘Valesca’ in comparison to cv. ‘Casino’. Based on densitometry analysis of bands, the accumulation of 47 kDa dehydrin in both of the investigated organs of the cv. ‘Valesca’ increased, while the amount of this dehydrin in the cv. ‘Casino’ significantly decreased (Figure 7).

### 3.7. Influence of Auxin Physiological Analogues on Protein Composition in the Oilseed Rape Cultivars ‘Casino’ and ‘Valesca’ during the Cold Acclimation Period

According to native PAGE data, the formation of approximately four (4) new proteins that were not typical for control in the terminal bud and up to six (6) proteins in the root collar of the cv. ‘Casino’ was induced by the compound TA-14 at the end of the cold acclimation period. Accordingly, the formation of approximately four new proteins not typical for control in each of the investigated organs, i.e., the terminal bud and root collar of the cv. ‘Valesca’ at the end of the cold acclimation period, was induced by the compound TA-14. The influence that was caused by the compound TA-12 on the formation of new proteins was weaker in both cultivars—one protein less in the terminal bud and two proteins less in the root collar—than that of the compound TA-14.

The accumulation of 42 kDa and 47 kDa polypeptides in the terminal bud of the cv. ‘Casino’ was influenced by both compounds. Notably, these polypeptides were detected in cv. ‘Valesca’ control plants at the end of the cold acclimation period. The formation of 40 kDa polypeptide in the root collar of cv. ‘Valesca’ was induced by the compound TA-14, and the formation of 81 kDa polypeptide in the root collar of the cv. ‘Casino’ was induced by both compounds. Further to that, the compounds TA-14 and TA-12 increased polypeptide 25 kDa accumulation and had an influence on the formation of 23 kDa polypeptide, that was not typical for control in organs that were important for wintering, in both oilseed rape cultivars.

### 3.8. Influence of Auxin-like Compounds on Protein Composition in the Oilseed Rape Cultivars ‘Casino’ and ‘Valesca’ during the Cold Acclimation Period

**Figure 8:**
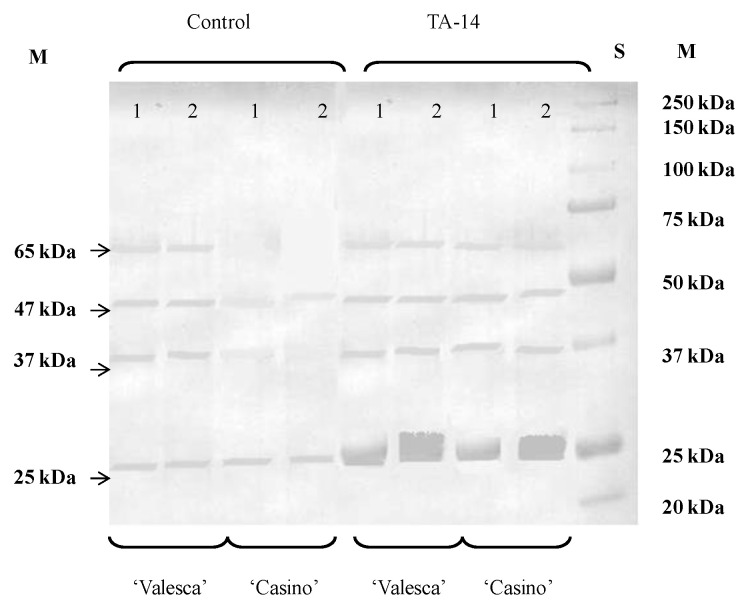
Specific influence of compound TA-14 on the accumulation of dehydrins in the terminal buds (1) and root collars (2) of the oilseed rape cultivars ‘Casino’ and ‘Valesca’ at the end of the cold acclimation period.

The formation and accumulation of 65 kDa and 47 kDa dehydrins which, as mentioned above, was detected in the cv. ‘Valesca’ during the entire cold acclimation period was induced by the compounds TA-14 and TA-12 in both organs of the cv. ‘Casino’. The accumulation of 25 kDa dehydrin in the organs of both cultivars was stimulated by auxin-like compounds (Figure 8).

### 3.9. Amount and Status of Phytohormone IAA and Peculiarities of Its Changes in the Oilseed Rape Cultivars Different for Their Resistance to Wintering during the Autumn Growth and at the End of the Cold Acclimation Period

#### 3.9.1. Amount and Status of Phytohormone IAA during the Autumn Growth Period

**Figure 9:**
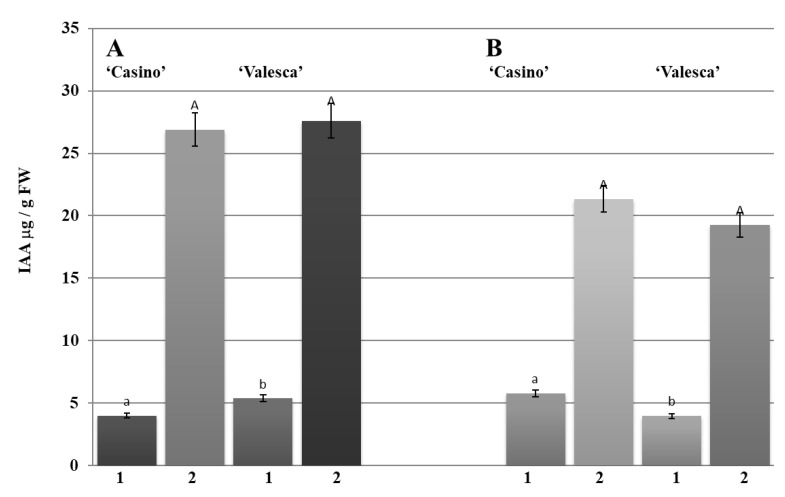
Amounts of IAA and IAA conjugates in the terminal buds (**A**) and root collars (**B**) of the oilseed rape cultivars during the autumn growth period: 1—free IAA; 2—IAA conjugates. There were four biological experiments with three replicates in each. Within each organ, different lowercase letters indicate significant differences in the mean amount of free IAA between the two oilseed rape cultivars. Within each organ, different uppercase letters indicate significant differences in the mean amount of IAA conjugates between the two oilseed rape cultivars. Bars are the means ± SD with different letters and are significantly different at *p* < 0.05 according to the Duncan test.

IAA was found in a free form and IAA conjugates formed in the investigated organs of both cultivars during the autumn growth period. The main amount of IAA was accumulated in the form of IAA conjugates. Our study showed that the amount of free IAA that was detected in the terminal bud of the cv. ‘Casino’ was 3.99 μg/g FW, while in ‘Valesca’ it was 5.36 μg/g FW. However, the mean amount of free IAA that was detected in the terminal bud of the more resistant to winter cv. ‘Valesca’ was 25% higher than in the cv. ‘Casino’ (Figure 9A).

Unlike in the terminal bud, 5.76 µg/g FW of free IAA was detected in the root collar of the cv. ‘Casino’ and in the cv. ‘Valesca’ 3.96 µg/g FW (Figure 9B). No significant differences were observed comparing the amounts of IAA conjugates in the investigated organs of either oilseed rape cultivars (Figure 9). The average amount of IAA conjugates that were detected in the terminal bud for cv. ‘Casino’ was 26.91 μg/g FW and for cv. ‘Valesca’ it was—27.61 µg/g FW. The average amount of IAA conjugates that were detected in the root collar in cv. ‘Casino’ was 21.34 μg/g FW, while in cv. ‘Valesca’ it was—19.26 µg/g FW.

Comparative analysis of the IAA conjugates, i.e., the quantities of IAA-esters and IAA-amides, showed that the major parts of the conjugates that were detected in the terminal buds of both cultivars were the IAA-amides, composing from 65% to 72% of the total amount of IAA conjugates. Meanwhile, higher amounts of IAA-esters, in comparison to IAA-amides, were found in the root collars.

#### 3.9.2. Amount and Status of IAA in the Early Period of Cold Acclimation

**Figure 10:**
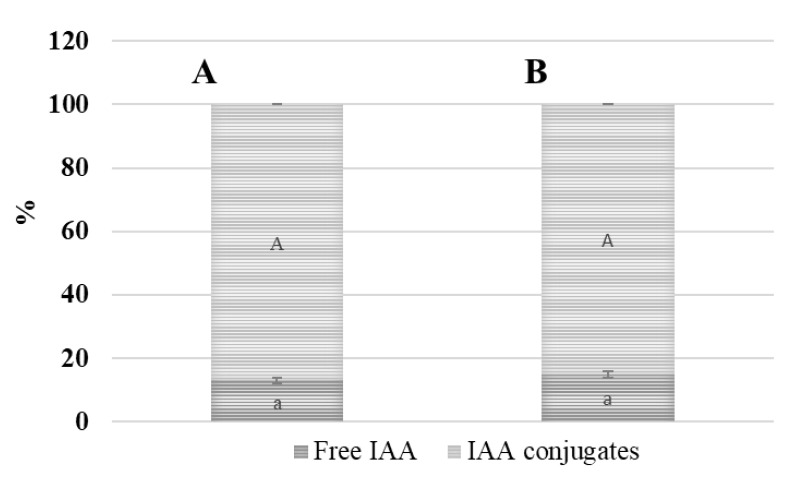
Status of the IAA fund in the terminal buds of the oilseed rape cultivars ‘Casino’ (**A**) and ‘Valesca’ (**B**) during the early period of cold acclimation. There were four biological experiments with three replicates in each. Different lowercase letters indicate significant differences in the mean percentage of free IAA between the two oilseed rape cultivars. Different uppercase letters indicate significant differences in the mean percentage of IAA conjugates between the two oilseed rape cultivars. Bars are the means ± SD with different letters and are significantly different at *p* < 0.05 according to the Duncan test.

The amount of free IAA remained similar in the terminal buds of both the investigated oilseed rape cultivars during the early period of cold acclimation compared with the autumn growth period. The physiologically active IAA in the terminal bud was composed about 15% of the total IAA fund in the early period of cold acclimation (Figure 10). The amount of the IAA conjugates in the terminal bud depended on the year that was investigated but fluctuated in the range from 25.80 to 30.30 μg/g FW in cv. ‘Casino’, and from 22.60 to 28.90 μg/g FW in cv. ‘Valesca’.

**Figure 11:**
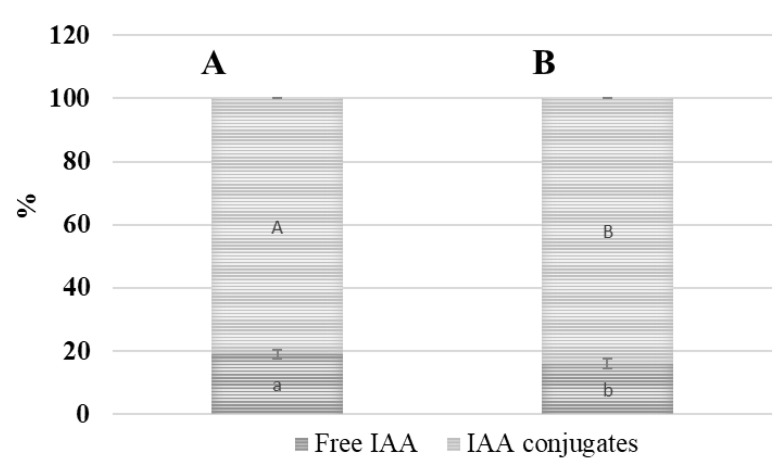
Status of the IAA fund in the root collars of the oilseed rape cultivars ‘Casino’ (**A**) and ‘Valesca’ (**B**) during the early period of cold acclimation. There were four biological experiments with three replicates in each. Different lowercase letters indicate significant differences in the mean percentage of free IAA between the two oilseed rape cultivars. Different uppercase letters indicate significant differences in the mean percentage of IAA conjugates between two oilseed rape cultivars. Bars are the means ± SD with different letters and are significantly different at *p* < 0.05 according to the Duncan test.

The amount of free IAA in the terminal buds of both cultivars increased on average by 30% in cv. ‘Valesca’ and only 11% in cv. ‘Casino’, compared to the autumn growth period. The amount of IAA conjugates in the root collar of the cv. ‘Casino’ was detected in the range from 23.90 μg/g FW and in cv. ‘Valesca’ from 32.05 μg/g FW. The amount of IAA conjugates in the root collar of the cv. ‘Valesca’ increased to 66.92% compared to the autumn period, while in cv. ‘Casino’ only 11.79%. Thus, the amount of IAA conjugates that were accumulated in the terminal bud and, especially, in the root collar of the more resistant cv. ‘Valesca’ significantly exceeded the amount that was found in the cv. ‘Casino’. The amount of free IAA in the root collars of both cultivars ranged from 16% to 19% of the total IAA fund (Figure 11).

Differences in the IAA amount and status that were detected among the organs of both cultivars were even more evident at the end of the cold acclimation period.

#### 3.9.3. Amount and Status of IAA in the Late Period of Cold Acclimation

An amount of free IAA was not detected in the terminal bud of the cv. ‘Valesca’ in the late period of oilseed rape cold acclimation. At the same time, an amount of free IAA, although in small quantities, i.e., 2.99 μg/g FW, was detected in plants of the cv. ‘Casino’. Quantities of IAA conjugates in the organs of both oilseed rape cultivars remained similar to the previously investigated acclimation period.

#### 3.9.4. Amount and Status of IAA at the End of the Cold Acclimation Period

**Figure 12:**
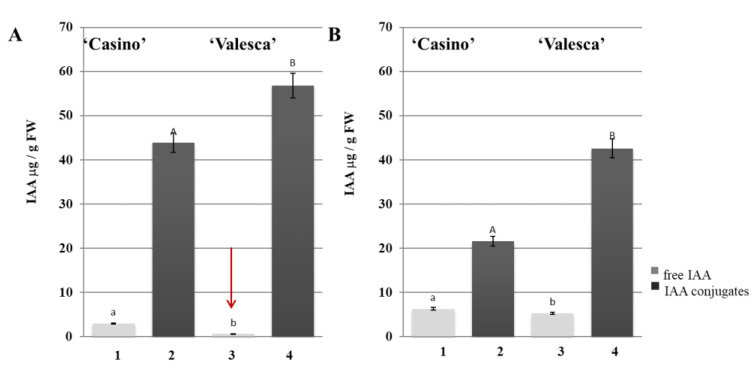
Transformation of the IAA fund and status in the terminal buds (**A**) and root collars (**B**) of the oilseed rape cultivars ‘Valesca’ and ‘Casino’ at the end of the cold acclimation period. There were four biological experiments with three replicates in each. Within each organ, different lowercase letters indicate significant differences in the mean amount of free IAA between the two oilseed rape cultivars. Within each organ, different uppercase letters indicate significant differences in the mean amount of IAA conjugates between the two oilseed rape cultivars. Bars are the means ± SD with different letters and are significantly different at *p* < 0.05 according to the Duncan test.

The amount and status of IAA that was detected in the terminal buds and root collars of the oilseed rape cultivars ‘Valesca’ and ‘Casino’ differed significantly in both the investigated organs at the end of the cold acclimation and transition to dormancy period (Figure 12).

Our results revealed that up to 3.00 µg/g FW of free IAA content in the cv. ‘Casino’ terminal bud was detected at the end of cold acclimation. At the same time, no free IAA was detected in the cv. ‘Valesca’ terminal bud (Figure 12A). The amount of free IAA detected in the root collar of cv. ‘Valesca’ was 23% lower compared to ‘Casino’ (Figure 12B).

**Figure 13:**
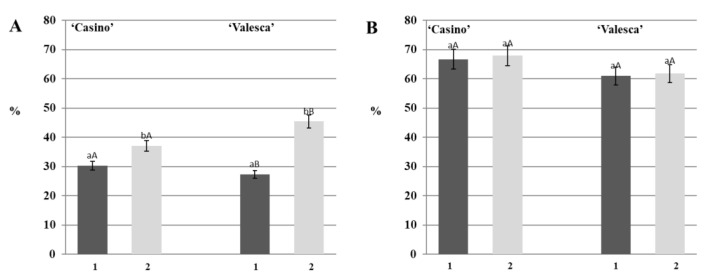
Percentage of IAA-esters from the total IAA conjugate fund in the terminal buds (**A**) and root collars (**B**) of the oilseed rapes cultivars ‘Casino’ and ‘Valesca’ during the autumn growth period (1) and at the end of the cold acclimation period (2). Within each cultivar in the same organ, different lowercase letters indicate significant differences in the mean percentage of IAA-esters from the total IAA conjugate in the two different growth and cold acclimation periods. Different uppercase letters indicate significant differences in the mean percentage of the IAA-esters from the total IAA conjugate in the same period between the two oilseed rape cultivars in the same organ. Bars are the means ± SD with different letters and are significantly different at *p* < 0.05 according to the Duncan test.

The intensive formation of IAA conjugates was detected at the end of the cold acclimation period. A two-times higher amount of IAA conjugates was found in the terminal buds and root collars than in the autumn growth period and the early cold acclimation period (Figure 13). The amount of IAA conjugates that were detected in the cv. ‘Valesca’ terminal bud was up to 56.83 μg/g FW, while in ‘Casino’ it was only 43.85 μg/g FW (Figure 12). To conclude, an around 23% higher amount of IAA conjugates was accumulated in the terminal bud of the more wintering-resistant cv. ‘Valesca’ at the end of cold accumulation period compared to the cv. ‘Casino’.

The amount of IAA conjugates in the root collar of the cv. ‘Valesca’ exceeded the amount that was detected in the cv. ‘Casino’ by about two times, i.e., the average detected amount was 42.50 µg/g FW in ‘Valesca’, while in ‘Casino’ it was only 21.60 µg/g FW.

After having compared the amounts of IAA-esters and IAA-amides type conjugates that were found in the organs of the investigated oilseed rape cultivars according to the duration of their cold acclimation, it became clear that the IAA-amide type conjugates had a clear tendency to decrease. It decreased in the terminal bud of the cv. ‘Valesca’ from 72.73% to 54.58% and in the cv. ‘Casino’ from 69.71% to 62.08% by the end of acclimation period. Thus, the proportion of the IAA-esters increased in the total fund of IAA conjugates at the end of cold acclimation period. The total amounts of the IAA-esters levels in the terminal bud of ‘Valesca’ increased by almost by 3.4 times, while in the less resistant to wintering ‘Casino’ only by 1.99 times (Figure 13A).

During the autumn growth and at the end of cold acclimation period, the amount of IAA-esters that were found in the root collars of both oilseed rape cultivars was similar, but not significantly different (Figure 13B), and an increase of IAA conjugates in cv. ‘Valesca’ was determined by IAA-amides.

#### 3.9.5. Final Identification of IAA and Metabolites in the Oilseed Rape Plants

Having compared TLC according to characteristics that were relevant to the assessment of indole compounds (color reactions, spot localization in plates, λ254 nm fluorescence, UV absorption spectra in the range from λ220 nm to λ320 nm with standards of synthetic IAA and its metabolites), three more indole compounds, apart of IAA, possessing all the characteristic properties, were detected. The detected IAA metabolites were preliminary identified as IAA-Asp, IAA-Glu, and IAA-Glc.

**Figure 14:**
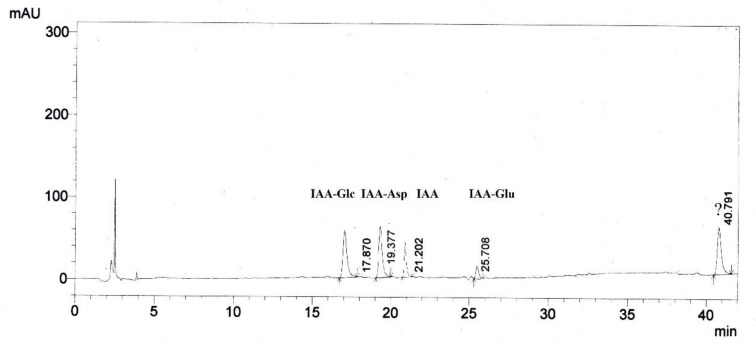
HPLC chromatogram of indole compounds (50 µM for each compound) monitored at 280 nm. Peak identities: retention time at 17.87 min—IAA-Glc; retention time at 19.37 min—IAA-Asp; retention time at 21.02 min—IAA; retention time at 25.71 min—IAA-Glu; retention time at 40.79 min—unknown indole compound.

The IAA and its compounds, i.e., IAA-amides—IAA-Asp, IAA-Glu, and the IAA-esters conjugate IAA-Glc, were finally identified by the HPLC method, comparing the absorption spectra of the eluted fractions from the chromatographic column and their retention time (Figure 14). Unfortunately, the fifth compound at the retention time eluted at 40.79 min could not be identified because it did not match any of the standards that we had. After analyzing the color reactions, λ254 nm fluorescence, UV absorption spectra in the range from λ220 nm to λ320, peak at λ280 nm, it is assumed to belong to the indole compounds.

### 3.10. Influence of the Auxin-like Compounds TA-12 and TA-14 on the Amount and Status of the IAA

Analyses of the effects of the auxin-like compounds TA-12 and TA-14 on the amount and status of IAA in the organs that were important for wintering in the cultivars ‘Casino’ and ‘Valesca’ revealed the effect of these compounds not only on the amount of free IAA, but also on the amount of IAA conjugates, depending on the duration of the acclimation period and the organs.

**Figure 15:**
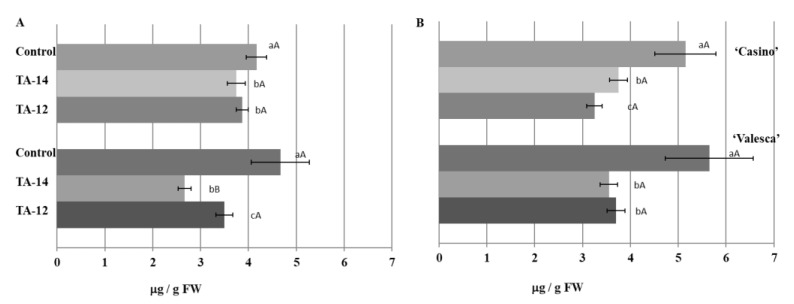
Influence of the compounds TA-14 and TA-12 on the amount of IAA in the terminal buds (**A**) and root collars (**B**) of the oilseed rape cultivars ‘Casino’ and ‘Valesca’ during the early period of cold acclimation. There were four biological experiments with three replicates in each. Within each cultivar in the same organ, different lowercase letters indicate significant differences in the mean amount of IAA between the control and two different treatments. Different uppercase letters indicate significant differences in the mean amount of IAA in the same organ between the two oilseed rape cultivars. Bars are the means ± SD with different letters and are significantly different at *p* < 0.05 according to the Duncan test.

Both of the investigated compounds, especially TA-14, had a distinct influence decreasing the IAA amount in the terminal bud of the winter-resistant oilseed rape cv. ‘Valesca’ already in the early period of cold acclimation. After TA-14, the free IAA amount decreased by 42.86%, and after TA-12 only by 24.99% (Figure 15A).

In the root collar of cv. ‘Casino’, the free IAA amount decreased by 27.18% after TA-14 and by 36.89% after TA-12. In the root collar of cv. ‘Valesca’, the free IAA amount decreased up to by 37.13% after applying of TA-14, and by 34.48% after TA-12 (Figure 15B).

After applying the compounds, especially the TA-14, the free IAA amount in the terminal bud of the cv. ‘Casino’ compared to the control also decreased at the end of the cold acclimation period. No statistically significant differences were registered under TA-12. No free IAA was detected in cv. ‘Valesca’ either in the control plants or after applying auxin-like compounds.

**Figure 16:**
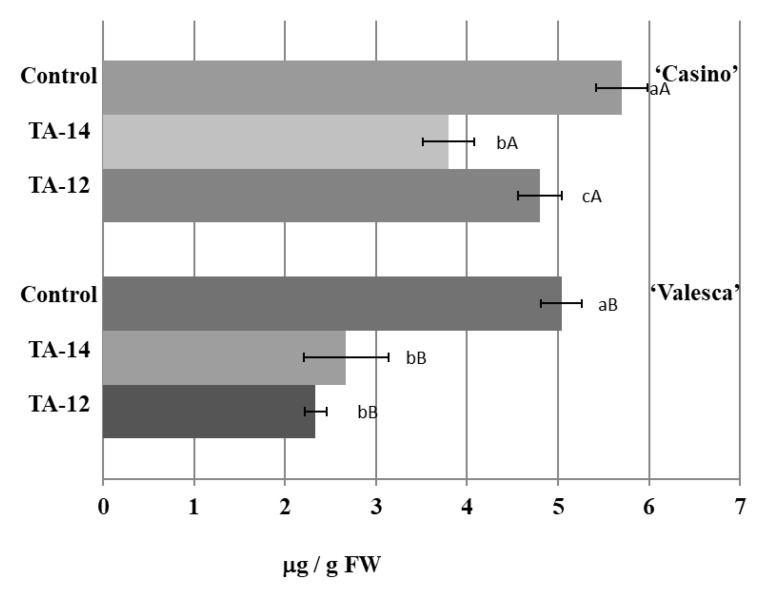
Influence of the compounds TA-14 and TA-12 on the amount of free IAA in the root collars of the oilseed rape cultivars ‘Casino’ and ‘Valesca’ at the end of the cold acclimation period. There were four biological experiments with three replicates in each. Within each cultivar, different lowercase letters indicate significant differences in the mean amount of IAA between the control and two different treatments. Different uppercase letters indicate significant differences in the mean amount of IAA between the two oilseed rape cultivars. Bars are the means ± SD with different letters and are significantly different at *p* < 0.05 according to the Duncan test.

In the root collar of the cv. ‘Valesca’, the free IAA amount decreased on average by 23.02% after applying TA-14 and by 37.05% after TA-12. In the root collar of the cv. ‘Casino’, the free IAA amount decreased on average by 21.89% after applying TA-14, and by 18.75% after TA-12, this was notably less compared to the influence of TA-14 (Figure 16).

**Figure 17:**
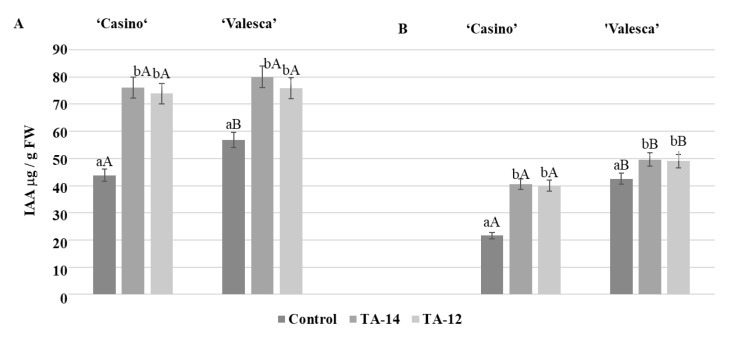
Influence of the compounds TA-14 and TA-12 on the amounts of IAA conjugates in the terminal buds (**A**) and root collars (**B**) of oilseed rape at the end of the cold acclimation period. There were four biological experiments with three replicates in each. Within each cultivar in the same organ, different lowercase letters indicate significant differences in the mean amount of IAA conjugates between the control and two different treatments. Different uppercase letters indicate significant differences in the mean amount of IAA conjugates in the same organ between the two oilseed rape cultivars. Bars are the means ± SD with different letters and are significantly different at *p* < 0.05 according to the Duncan test.

The intensive formation of IAA conjugates was detected at the end of the cold acclimation period after treatment with TA-14 and TA-12 (Figure 17).

The amount of IAA conjugates that were detected in the cv. ‘Valesca’ terminal bud under TA-14 was 80.01 μg/g FW, while under TA-12 it was 75.91 μg/g FW. Meanwhile, in cv. ‘Casino’, the auxin-like compounds increased the amount of IAA conjugates more notably, i.e., IAA conjugate values were similar to cv. ‘Valesca’ in the terminal bud (Figure 17). A twice higher amount of IAA conjugates was found in the cv. ‘Casino’ root collar under auxin-like compounds at the end of cold acclimation, while the amount of IAA conjugates increased much less—up to 5.90%—in the more resistant for cold oilseed rape cv. ‘Valesca’ (Figure 17).

### 3.11. Influence of Auxin-like Compounds on Biometric Parameters and Overwintering

**Figure 18:**
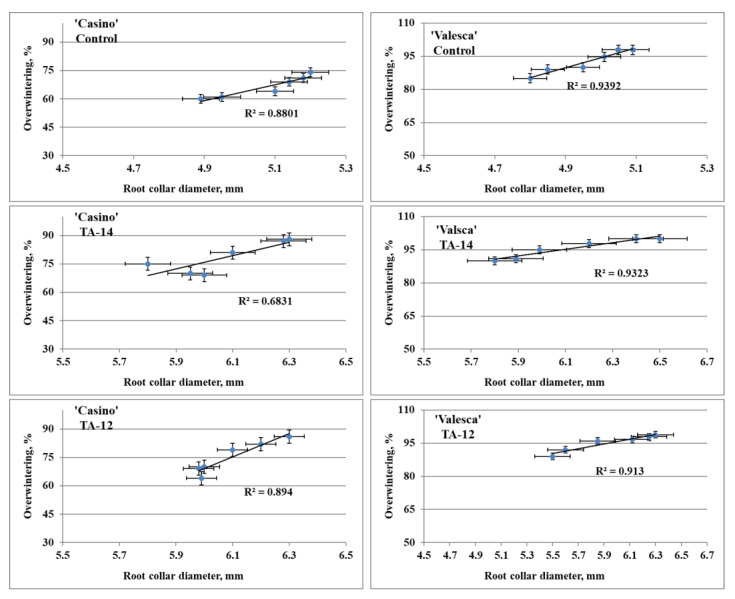
Dependence of oilseed rape overwintering on the diameter of the root collars. Both horizontal and vertical bars indicate the means ± SD, data are from four biological experiments with three replicates in each, 20 root collars per replicate.

Results from investigations in the small experimental fields showed that after applying TA-12 and TA-14 compounds, oilseed rapes overwintered better. Correlation-regression analysis of the data that were obtained showed that the overwintering of ‘Valesca’ and ‘Casino’ plants directly depended on the diameter of the root collar. Data regression analysis showed a strong dependence of plant overwintering on the diameter of the root collar. Having calculated the dependence of plant overwintering on the diameter of the root collar, it was determined that the diameter of the root collar both in the control and treated plants had a large influence on the overwintering of the oilseed rapes. This dependence was indicated by the determination coefficient R^2^ (Figure 18).

The tested auxin-like compounds showed significant stimulating effects not only on the oilseed rape root collars, but also on the terminal bud diameters. The analyzed terminal bud diameters were significantly influenced by TA-14 and TA-12 in both the tested cultivars (Table 4). However, no difference was found between the tested cultivars under TA-14 and TA-12 treatments.

## 4. Discussion

Plant adaptability and resistance to wintering are among the most relevant plant physiological-biochemical and biotechnological issues that should be understood, first of all in the theoretical aspect, i.e., through clarifying the systems determining plant resistance and adaptability, as well as the links in metabolic processes [53,54,55]. Moreover, through understanding these mechanisms, it is possible to manage wintering processes with biotechnological tools.

It has been widely reported, in order to reveal the preparation of the cold acclimation mechanisms, great attention should be paid to changes in membranes and their functions [11,13,14,56]. However, while it is recognized that proteins are key structural and functional elements within the cell, such as structural components of membranes, transporters, enzymes, and receptors, influencing and determining a variety of metabolic processes within the cell, including growth and development processes and cold resistance [57,58,59,60], till now, their metabolic processes, compositional changes, and relationships with plant resistance and preparation for wintering have not been fully investigated and disclosed. Experiments, especially on herbaceous plants, are usually carried out by simulating the process of cold acclimation, i.e., by changing the temperature and the duration of the photoperiod under control conditions [29,47,61]. Previous studies have been shown changes in herbaceous plant proteins under natural conditions, including common wheat [62] and alfalfa [63,64]. When analyzing the role of phytohormones in the plant cold acclimation process, the main focus has been on ABA [15,16,19], but unfortunately little attention has been paid to IAA [23,65,66,67] and control of the cold acclimation period with auxin-like compounds.

In the current study, in order to reveal changes in the protein composition and IAA content and status during the period of cold acclimation and possible links between cold acclimation and the protein composition and IAA, we chose as test objects two oilseed rape cultivars that differ in terms of resistance for wintering, specifically cv. ‘Casino’ (less resistance for wintering in the Lithuanian climate condition) and cv. ‘Valesca’ (more resistance for wintering in the Lithuanian climate condition). Moreover, to reveal the IAA level, the protein composition and to characterize the possible implementation of auxin during the cold acclimation processes, that extends over the cold acclimation period, externally applied auxin-like compounds were used.

### 4.1. Relationship between Auxin-like Compounds TA-12 and TA-14 and Protein Metabolism Reorganization and Oilseed Rape Cold Acclimation

Native electrophoresis, denaturing SDS-PAGE, and immunoblotting methods were performed to confirm changes in the protein composition and their metabolic processes in the winter oilseed rape cultivars. In contrast to the work of other authors [47,61], in which only the soluble protein fractions were analyzed, in the current study we also analyzed changes in the proteins from the cell membrane component during the oilseed rape natural cold acclimation. It had been reported that changes in the protein composition from the cell membranes during the period of cold acclimation in the organs which are important for wintering in herbaceous plants (the terminal bud and root collar) need to be analyzed due to changes in the condition and functional properties of membranes [11,56,68]. In addition, proteins from the cell membrane are essential for the study of phytohormones and their interaction with receptors [57,60,69].

The analysis of the protein number during the autumn growth period and the cold acclimation period shows that it is volatile and varies according to the duration of acclimation (Figure 1, Figure 2, Figure 3 and Figure 4). In both the tested cultivars, the terminal buds and root collars underwent intense protein metabolism during the period of hardening, leading to the disappearance of proteins that are characteristic to the period of growth and the de novo formation and accumulation of proteins (Table 1). The composition of the ESP fractions was further modified in the early cold acclimation period and the HSP fractions in the later periods (Figure 5 and Figure 6). In addition, changes in the protein composition of the more resistant cv. ‘Valesca’ were more intensive than in the less resistant ‘Casino’, especially in the root collar. Thus, after the protein analysis of the two investigated oilseed rape cultivars prepared for wintering by native PAGE, it was revealed that nine (9) proteins that were detected in the ESP and HSP terminal bud fractions of the cv. ‘Valesca’ were distinctive and unique to this cultivar only. Therefore, an assumption could be made that the presence of these proteins can be attributed to the ability of the oilseed rape cv. ‘Valesca’ for better acclimation and preparation for wintering, i.e., for cold acclimation and the dormancy period. Vice versa, the lack of the mentioned proteins can be associated with the lower wintering resistance of cv. ‘Casino’.

The studies indicate that the formation and accumulation of low and medium molecular weight proteins in various plant organs that are important for wintering is associated with cold acclimation and wintering resistance [24,62,70]. The functions of high molecular weight proteins may be otherwise [47,71,72]. The fact that a higher amount and diversity of proteins was detected in the organs of the more resistant cv. ‘Valesca’, compared to the less resistant cv. ‘Casino’, supposes an assumption that proteins with different functions may be of greater variety and this, in turn, may influence cold acclimation and successful wintering during the plant dormancy period. Given the significantly slower conversion of both protein fractions of the oilseed rape root collar proteins during the cold acclimation period, in particular of the HSP fraction, it can be definitely confirmed that the role of the oilseed rape root collar in wintering resistance is important [7,37,73]. In addition, after treatment with auxin-like compounds, data from the native PAGE electrophoresis result showed a 10–12% increase in the protein content of the terminal bud of the ‘Casino’ cultivar of oilseed rape (Figure 5) and 16–18% of the root collar (Figure 6).

The fact that the more winter-resistant cv. ‘Valesca’, compared to the less winter-resistant cv. ‘Casino’, showed a higher number of proteins in the studied organs, and that their composition was more diverse, suggests that the variety of proteins with different functions may be greater, which in turn may have a significant impact on plant cold acclimation, successful overwintering, and maintenance of the dormancy period. These results correlate with data from other authors [26,42,62,74] showing that during the period of cold acclimation, an increase in protein number is a characteristic biochemical process that is associated with the wintering resistance of plants. The slow dynamics of the changes in the protein amount in the root collar of the cv. ‘Casino’ suggests that this rapeseed cultivar is not able to completely restructure its metabolic processes during the cold acclimation process, which may be associated with the lower survival rate in winter.

The data that were obtained by the Native PAGE method on the protein composition of the ESP and HSP fractions and their number in the terminal buds and root collars of the oilseed rape cultivars with different resistance to wintering led to the question of how the composition of both cultivars of polypeptides and thermostable proteins (dehydrins) differ.

The distinctive specific polypeptides for the investigated different oilseed rape cultivars were detected (Table 2). The cv. ‘Valesca’ was distinctive by its high number of specific polypeptides (interval from 8 kDa to 65 kDa), especially in the HSP fraction in both of the investigated organs. It was proven that the more resistant to wintering cv. ‘Valesca’ was characterized by a wider range of polypeptides in the composition in both organs at this level of investigation.

The summarized results showed that some of the polypeptides that were detected in the oilseed rapes were also common to other wintering plants [25,62,75,76].

It was found that a higher number of thermostable proteins accumulated in the terminal bud and root collar (ESP fraction) of the more resistant to winter oilseed rape cv. ‘Valesca’, compared to the less resistant cv. ‘Casino’, during the autumn growth as well as at the end of the cold acclimation period. At the end of the cold acclimation period, the cv. ‘Valesca’ had a higher number of thermostable proteins, especially in the root collar (Table 2 and Table 3). The molecular weights of the thermostable polypeptides that were found in the investigated organs of the oilseed rapes were detected in the range from 10 to 150 kDa. They correlate with the molecular weights of polypeptides determining wintering resistance in other overwintering plants (barley, wheat, rhododendron, etc.) [18,25,47,62,77,78]. Based on the results of the thermostable protein composition, it could be concluded that the 10; 12; 18; 37; 47; 50; 65; 69; 75; 84; 87; 91; 150 kDa thermostable polypeptides that were found in the tested organs of the cv. ‘Valesca’ could be associated with the wintering resistance of that cultivar, whereas the absence of these thermostable polypeptides in the organs of the cv. ‘Casino’ may be related to its lack of ability to acclimate and its worse wintering (Figure 18). The results that were obtained suggested that in order to maintain proper metabolic processes in the investigated oilseed rape cultivars for their preparation to winter, an important role was attributed not only for the polypeptides that were found during the entire acclimation period, but also for the newly formed polypeptides.

A previous study has shown that thermostable proteins-dehydrins determine the winter resistance of plants [9,15,17,25,45,79]. In the present study, we identified four dehydrins. In the cv. ‘Valesca’, 37; 47; 65 kDa dehydrins were identified during the autumn growth period and an additional 25 kDa dehydrin was identified at the end of cold acclimation (not characteristic of autumn growth) (Figure 7). After treatment with the auxin-like compounds, the formation of 65 and 47 kDa dehydrins was observed in the cv. ‘Casino’ (Figure 8).

It is supposed that the 25; 37; 47 kDa dehydrins could serve as protein-markers for assessing acclimation and cold resistance in the *Brassicaceae* family. The results that were obtained suggest that the lower wintering resistance of ‘Casino’ plants compared with ‘Valesca’ plants may be caused by less accumulation of 37 and 47 kDa dehydrins. These results support the verdict regarding dehydrins in *Brassicaceae* family plants [61]. However, the available data have extended the knowledge of the existence of other dehydrins in plants of the *Brassicaceae* family.

Thus, the results show that changes in proteins in soluble and membranes or membranes surrounding structural cell components are one of the key biochemical-molecular changes occurring during the adaptation for cold acclimation and preparation for wintering process. This is confirmed by the data from the experiments that were carried out with the auxin-like compounds. In our previous study, it was shown that after treatment with the auxin-like compounds TA-12 and TA-14, the winter resistance of the studied oilseed rape cv. ‘Valesca’ increased [32]. In the current study, we extend knowledge about the integration of auxin-like compounds into cell systems during the preparation for wintering. The positive effects of these compounds on the formation and accumulation of specific dehydrins in the organs of the oilseed rapes show that not only changes in the photoperiod and temperature have an effect, but also the hormonal system has the ability to participate in the cold acclimation process.

### 4.2. The Influence of Auxin-like Compounds TA-12 and TA-14 on IAA Metabolism and Their Relationship with Oilseed Rape Cold Acclimation

In order to reveal the peculiarities of IAA metabolism processes, and thus the peculiarities of its possible involvement in adaptive processes and the links with wintering resistance, we analyzed the amount of IAA, its status, and the composition of the fund according to the wintering resistance in the terminal buds and root collars of the different rapeseed cultivars during the autumn growth period, subsequent cold acclimation periods, and at the end of cold acclimation.

Our studies showed that there were intense changes in the IAA levels and status through all cold acclimation periods (Figure 9, Figure 10, Figure 11, Figure 12 and Figure 13). As the free IAA decreased, the amount of IAA conjugates increased in parallel, and the ratio of IAA-esters to IAA-amides changed. However, although both cultivars shared the same trend in terms of IAA levels and status, the results of the comparative analysis highlighted the differences between the cultivars and organs.

The present study showed that nearly 16% of the total IAA fund was composed by free IAA in a physiologically active form in the terminal buds of both the investigated cultivars during the autumn growth period. More diverse results were obtained in the root collar: free IAA in cv. ‘Valesca’ consisted of up to 15%, while it was 21% in cv. ‘Casino’. This was in agreement with the normal ratio among IAA and IAA conjugates in vegetative organs of various plants during the growth period [80,81,82,83]. It was found that in the terminal bud of the cv. ‘Valesca’, there was a significantly reduced amount of free IAA in the early cold acclimation period compared to the autumn growth stage, and free IAA were not detected at the end of the cold acclimation period. Meanwhile, in the less resistant cv. ‘Casino’, free IAA were reduced by only 27% compared to the autumn growth period (Figure 12A). The amount of free IAA in the root collars at the end of the cold acclimation period remained similar in both the tested cultivars but was 28% lower in the cv. ‘Valesca’ than in the cv. ‘Casino’ (Figure 12B). At the end of the cold acclimation period, the amount of IAA-esters in the terminal buds increased significantly, especially in the cv. ‘Valesca’ (Figure 13A), while in the root collars of both cultivars it remained similar and sufficiently high throughout the period of cold acclimation (Figure 13B). Therefore, the increase in IAA conjugates was due to the increase in IAA-amides. These data correlate with data from other authors that indicate that the amount of free IAA decreases during tempering at low temperatures and the amount of IAR conjugates increases [84,85]. IAA conjugates are recognized to be involved in controlling the level of IAA homeostasis in a cell [81,82,83,86] and are a major source of free IAA.

As expected, we identified IAA-amides—IAA-Asp, IAA-Glu, and the IAA ester IAA-Glc (Figure 15), which are conjugates that are characteristic for monocotyledonous and dicotyledonous plants [49,81,87,88] as well as for *Arabidopsis* [80,82,89]. These commonly known IAA metabolites are generally recognized as the compounds maintaining IAA homeostasis [81,82]. The data that were collected about the absence of free IAA in the terminal bud of the more resistant cv. ‘Valesca’ and the higher amount of IAA conjugates (IAA-amides and IAA-esters) in both organs (changed ratio of IAA-esters and IAR-amides) at the end of the cold acclimation period, compared to the less resistant cv. ‘Casino’, suggest an assumption that a decline in free IAA and IAA conjugates in the organs that are important for wintering thereby results in their ability for cold acclimation. In order to confirm this assumption, investigations using auxin-like compounds TA-12 and TA-14 were carried out. It was revealed that these auxin-like compounds have the following influences: (a) decrease the level of free IAA, especially in both of the investigated organs of the resistant cv. ‘Valesca’ during the early cold acclimation period (Figure 15); (b) change the ratio of IAA-esters and IAA-amides; (c) form IAA metabolites. The results that were obtained suggest that auxin-like compounds may affect not only the composition of proteins (as was discussed in the previous section) and the amount of physiologically active IAA, but also the metabolic processes in the organs that are important during preparation for the cold acclimation period.

Preliminary data that were collected suggest that the auxin-like compounds TA-12 and TA-14 have a tendency to affect the ratio of IAA-ester and IAA-amide types of conjugates in the terminal buds and root collars of both of the investigated oilseed rape cultivars.

These data expand the understanding of the potential role of the IAA status (IAA conjugated forms) in cold acclimation and wintering resistance [84,85]. Our results suggest that IAA conjugates are involved in controlling the level of IAA intracellular homeostasis [81,82,83,86], this being a major source of free IAA and this may affect the renewal processes of growth and development in spring [90].

A number of authors have found that the actions of various stressors inhibit or inversely induce the formation and accumulation of proteins that are involved in IAA metabolism and the mechanism of its function. They have also provided data on the early genes of the auxin response *Aux/IAA*, *GH3*, *SAUR* [81,91] encoding the low- and medium-molecular weight proteins Aux/IAA, GH3, and SAURS, which are localized in the cytoplasm and nucleus. Keeping in mind that both in the control plants and after treatment with auxin-like compounds during the period of cold acclimation and preparation for wintering, there is a significant change in the protein composition and IAA content during the transition to the dormancy period in the more resistant cv. ‘Valesca’. It can be stated that there is a close relationship between the transformation of the protein composition and the involvement of the phytohormone IAA in the adaptive processes for unfavorable temperatures.

## 5. Conclusions

Auxin-like compounds—TA-14 and TA-12—influence the rearrangement of protein composition in the overwintering organs of the investigated oilseed rape cultivars in natural field conditions. They stimulate the de novo formation of proteins, especially cold resistance-determining proteins (dehydrins). They also affect the amount, metabolism, and status of IAA; the amount of free IAA is decreased, and the amount of IAA-conjugates is increased. This work was the first investigating the changes in the composition of the protein fractions of the overwintering organs (terminal bud and root collar) of oilseed rape that were grown in experimental fields through all cold acclimation periods in preparation for wintering. In this work, not only general changes in the protein composition but also specific in the protein fractions of the different organs of the two oilseed rape cultivars during cold acclimation period were elucidated.

The data of our study show that the transformation of the protein composition, the endogenous IAA amount, and the status are the main biochemical and physiological factors influencing the wintering resistance of oilseed rapes.

## Figures and Tables

**Table 1:** Characteristics of protein metabolism at the end of the cold acclimation period.

Organ	Protein	Oilseed Rape Cultivars	
		‘Valesca’	‘Casino’
Terminal bud	Disappeared	25.80 ± 0.20 aA	31.15 ± 2.15 aB
	Newly formed	37.25 ± 2.25 bA	40.63 ± 3.13 bA
	Remaining the same	36.95 ± 2.05 cA	28.23 ± 0.98 cB
Root collar	Disappeared	16.91 ± 2.09 aA	25.21 ± 5.40 aB
	Newly formed	38.12 ± 2.52 bA	32.25 ± 1.25 bB
	Remaining the same	45.03 ± 0.13 cA	42.50 ± 1.15 cB

There were four biological experiments with three replicates in each. Within each cultivar in the same organ, different lowercase letters indicate significant differences in the mean percentage of protein metabolism changes that were classified according to three characteristics. Different uppercase letters indicate significant differences in the mean percentage of protein metabolism changes in the same characteristic between the two rapeseed cultivars in the same organ. Bars are the means ± SD with different letters and are significantly different at *p* < 0.05 according to the Duncan test.

**Table 2:** Specific polypeptides of the oilseed rape cultivars ‘Casino’ and ‘Valesca’ at the end of the cold acclimation period.

Oilseed Rape Cultivars	Organs	Fraction	
		ESP, kDa	HSP, kDa
**‘Casino’**	Terminal bud	89	96; 20
	Root collar	46; 28	32
**‘Valesca’**	Terminal bud	119; 48; 47; 43; 42	91; 59; 49; 39; 37; 28; 24; 18; 10; 8
	Root collar	250; 55; 37; 17; 10; 8; 9	150; 75; 65; 24; 12; 10

Data represent four biological experiments with three replicates in each.

**Table 3:** Percentage of thermostable proteins in the ESP fraction of the terminal buds and root collars of the oilseed rape cultivars ‘Casino’ and ‘Valesca’ cultivars during the autumn growth and at the end of the cold acclimation periods.

Growth and Cold Acclimation Periods	Organs	Oilseed Rape Cultivars	
		‘Casino’	‘Valesca’
**Autumn growth**	Terminal bud	9.53 ± 2.83 aA	16.73 ± 2.06 aB
	Root collar	12.60 ± 1.06 cA	16.73 ± 2.92 cB
**The end of cold acclimation**	Terminal bud	35.76 ± 3.43 bA	48.19 ± 4.44 bB
	Root collar	37.45 ± 1.65 dA	51.78 ± 7.61 dB

There were four biological experiments with three replicates in each. Within each cultivar in the same organ, different lowercase letters indicate significant differences in the mean percentage of thermostable proteins during the growth and cold acclimation periods. Different uppercase letters indicate significant differences in the mean percentage of thermostable proteins in the same period between the two oilseed rape cultivars at the same organ. Bars are the means ± SD with different letters and are significantly different at *p* < 0.05 according to the Duncan test.

**Table 4:** Effect of auxin-like compounds on the terminal bud diameters of the oilseed rape cultivars ‘Casino’ and ‘Valesca’ at the end of the cold acclimation period.

Test Variant	‘Casino’	‘Valesca’
Control	4.35 ± 0.13 aA	4.76 ± 0.18 cB
TA-14	6.21 ± 0.24 bA	6.40 ± 0.30 dA
TA-12	6.23 ± 0.23 bA	6.33 ± 0.50 dA

Data are from four biological experiments with three replicates in each, 20 terminal buds per replicate. Within each cultivar, different lowercase letters indicate significant differences in mean diameters of terminal buds between the control and two different treatments. Different uppercase letters indicate significant differences in the mean diameters of the terminal buds between the two rapeseed cultivars in the same test variant. Terminal bud diameter (mm) ± SD with different letters are significantly different at *p* < 0.05 according to the Duncan test.

## Data Availability

The data supporting the reported results can be found in the scientific reports of the Laboratory of Plant Physiology of the Institute of Botany of Nature Research Centre, where archived datasets that were generated during the study are included.
